# The late phase of sepsis is characterized by an increased microbiological burden and death rate

**DOI:** 10.1186/cc10332

**Published:** 2011-07-28

**Authors:** Gordon P Otto, Maik Sossdorf, Ralf A Claus, Jürgen Rödel, Katja Menge, Konrad Reinhart, Michael Bauer, Niels C Riedemann

**Affiliations:** 1 Clinic for Anesthesiology and Intensive Care, Jena University Hospital, Erlanger Allee 101, 07747 Jena, Germany; 2 Institute of Medical Microbiology, Jena University Hospital, Erlanger Allee 101, 07747 Jena, Germany

## Abstract

**Introduction:**

Recent models capturing the pathophysiology of sepsis and *ex-vivo *data from patients are speculating about immunosuppression in the so-called late phase of sepsis. Clinical data regarding survival and microbiological burden are missing. The aim of this study was to determine the clinical significance of the 'late phase' of sepsis with respect to overall survival and occurrence of microbiological findings.

**Methods:**

In a retrospective trial, 16,041 patient charts from a university intensive care unit were screened, and 999 patients with severe sepsis or septic shock were identified. Three phases were established according to the mortality peaks which were separated by two distinct nadirs: phase I (days 1 to 5), phase II (days 6 to 15) and phase III (days 16 to 150). Patients were analyzed for outcome, SOFA scores, procalcitonin levels, antimicrobial treatment, dialysis, mechanical ventilation and results of blood cultures during their hospital stay.

**Results:**

Out of 999 enrolled patients, 308 died during the course of sepsis presenting a characteristic mortality rate (30.8%) with three distinct mortality peaks (at days 2, 7 and 17). Overall 36.7% of all deaths occurred in the early phase (phase I) and 63.3% during the later phases (phase II + III). In total 2,117 blood cultures were drawn. In phase I, 882 blood cultures were drawn, representing a sampling rate of 88% with a positive rate of 14.9%. In phase II, 461 samples were taken, indicating a sampling rate of 52% and a positive rate of 11.3%. Within phase III, 524 samples were obtained representing a sampling rate of 66% with a positive rate of 15.3%, which was significantly higher compared to the positive rate of phase II and similar to phase I. In particular, the rate of typically opportunistic bacteria increased significantly from 9% in phase I up to 18% in phase III. The same is true for *Candida *spp. (phase I 13%, phase III 30%).

**Conclusions:**

The later phase of sepsis is associated with a significant re-increase of positive blood culture results, especially regarding opportunistic bacteria and fungi. These observations warrant further studies focusing on the underlying mechanisms resulting in this outcome burden in the later phase of sepsis.

## Introduction

Despite aggressive supportive care to improve treatment options and outcome, sepsis and its sequelae remain a leading cause of death in intensive care units [1]. Extensive studies investigating the host responses during sepsis have revealed that the late phase of sepsis is dominated by a status of immune suppression with respect to missing or widespread depressed innate, as well as adaptive, immune defense mechanisms. Since various authors introduced the intriguing model of a phase-dependent variation of immune activity [2-4], the concept of an anergic immune system is held responsible as a principal mechanism and cause of death in patients with sepsis [5,6]. However, the concept itself, but more the impact of sequential hyper- and hypo-inflammatory phases, has been discussed controversially [7-9]. The lack of tools assessing the individual immune status of patients at the bedside and the complex pathophysiological processes, which are often overlapping, have so far limited the meaning of such concepts describing hyper- and hypo-inflammatory immune phases for the clinician. Confirmatory studies demonstrating the clinical significance, especially of the so-called late phase in sepsis, are largely missing. We, therefore, thought to investigate whether a so-called late phase during the course of sepsis would be accompanied by higher death rates and higher rates of positive blood culture results, especially by typically opportunistic bacteria. The latter might be interpreted as a hint for an underlying immune suppressive status, which has been suggested by many studies investigating separate immune defense mechanisms in patients suffering from sepsis [2,3,6,9].

## Materials and methods

To clarify this question we used a large database of patient records from daily practice and performed a retrospective trial enrolling patients admitted from 1 January 2006 to 31 December 2009 to the intensive care unit of the Jena university hospital. The study was approved by the institutional review board of Friedrich Schiller University Hospital, Jena, Germany (3080-03/11). Informed consent was waived due to the anonymous nature of the analysis. A total of 16,041 patients were screened according to the criteria of the American College of Chest Physicians/Society of Critical Care Medicine (ACCP/SCCM) for severe sepsis or septic shock [10,11]. We identified 999 patients qualifying for severe sepsis or septic shock and evaluated demographics, clinical characteristics, disease severity measured by SOFA score [12,13], procalcitonin levels (PCT), requirement of renal replacement therapy and mechanical ventilation, and outcome of patients, as well as the results of microbiological blood cultures and antimicrobial treatment.

By the observed trajectory of mortality, periods with different mortality rates were identified and classified by the nadirs, yielding in the definition of three distinct phases: Phase I from Day 1 until Day 5, phase II from Day 6 until Day 15 and phase III from Day 16 until the end of the observation period at Day 150. To investigate the relevance of the various phases, analyses of all obtained blood cultures with respect to their date of sampling during patients' hospital stay were performed. In addition, samples taken 10 days prior to clinical diagnosis of sepsis were included and this time period was defined as phase 0 (Day -10 to Day -1). Coagulase negative staphylococci (CNS) were interpreted as skin contamination of no importance and presented separately. Furthermore, epidemiology of microorganisms in the different phases was analyzed. Beside the total number of positive findings, the relative numbers of drawn blood cultures in relation to the number of patients alive during that period and, in particular, the rate of positive findings per obtained samples during the corresponding phase were also analyzed. Additionally, the practice of antimicrobial treatment and clinical characteristics of patients were studied dependent of the defined phases and dependent of the results from the obtained blood cultures at the day of sampling.

### Statistics

All data are reported as relative numbers, including original absolute numbers. A Chi-square test was used for comparisons of categorical data. Continuous data across multiple groups were compared by ANOVA testing with Bonferroni adjustment. *P-*values < 0.05 were considered significant. Data were analyzed using SPSS software (version 13) from SPSS Inc. (Chicago, IL, USA).

## Results

### Demographics, clinical characteristics and outcome

**Figure 1:**
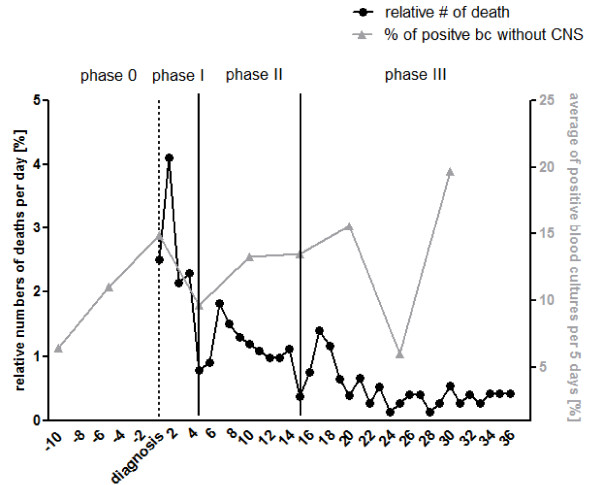
**Distribution of non survivors and positive blood cultures during sepsis**. Relative numbers of deaths per day from 999 patients with severe sepsis or septic shock according to ACCP/SCCM criteria are shown from the day of onset/diagnosis until observation Day 36. Three phases were defined, characterized by the nadir at Day 5 and Day 15. Also, the average rates of positive blood cultures without CNS in a five-day period with respect to sampling times are shown. bc, blood cultures; CNS, Coagulase negative staphylococci; #, numbers; %, relative number.

An overview of the demographics and clinical characteristics from the identified patients with severe sepsis or septic shock is given in Table 1. Out of these 999 patients, 308 died during the course of severe sepsis or septic shock. The outcome data plotted from first onset of sepsis until Day 36 of these enrolled patients were characterized by three peaks of increased mortality rates. The first one occurred at Day 2 after reported diagnosis, the second one at Day 7 and the last one at around Day 17 (Figure 1). In agreement with the addressed concept of a late phase of sepsis, the second and third maximum values might pertain to this phase of sepsis. The further analyses were strictly performed according to the defined phases by the observed nadirs of mortality rate (phase I: Day 1 until Day 5, phase II: Day 6 until Day 15, phase III: Day 16 until the end of the observation period at Day 150).

**Table 1:** Demographics and clinical characteristics of enrolled patients with severe sepsis or septic shock

Demographics and clinical characteristics	
Gender, male/female, #	660/339
Age (years), median (IQR)	67 (56 to 75)
BMI (kg/m^2^), median (IQR)	26.3 (23.9 to 29.6)
APACHE II Score at admission, median (IQR)	14 (11 to 19)
Type of ICU admission	
*Medical, # (%)*	410 (41)
*Surgical, # (%)*	589 (59)
Co-morbid conditions, # (%)	
*Hypertension*	533 (53)
*Diabetes*	305 (31)
*Cancer*	272 (27)
*Intestinal ischemia*	96 (10)
*Liver failure*	78 (8)

Characteristics of patients enrolled. BMI, body mass index; APACHE II, Acute Physiology and Chronic Health Evaluation II score was calculated at ICU admission; LOS, length of stay; IQR, interquartile range; #, numbers; %, relative number.

Out of all deaths, 36.7% occurred before the end of Day 5 during phase I and 63.3% (30.8% phase II and 32.5% phase III) during the following period (phase I vs. phase II + III *P *≤ 0.01; χ^2 ^test) (Table 2). The mortality rate of the later phase was 22% (10.7% phase II and 12.6% phase III) - significantly higher than in the early phase I with 11.3% (phase I vs. phase II + III *P *≤ 0.01; χ^2 ^test). With regard to these numbers, the later phases of sepsis, phase II and, especially, phase III, demonstrated a broad clinical significance.

**Table 2:** Characteristics of phase-dependent outcome and microbiological diagnosis

phases in relation to diagnosis	phase 0	phase I	phase II	phase III
days prior to diagnosis or during sepsis	Day -10 to Day -1	Day 1 to Day 5	Day 6 to Day 15	Day 16 to Day 150
# of patients alive at onset of phase, #	999	999	886	791
# of non survivors during phase, #		113	95	100
relative # of non survivors wrt all non survivors, # (%)		36.7 (113/308)	30.8 (95/308)	32.5 (100/308)
relative numbers of non survivors during phase, # (%)		11.3 (113/999)	10.7 (95/886)	12.6 (100/791)
# of drawn bc during phase, #	250	882	461	524
relative # of bc per patient alive, # (%)	25.0 (250/999)§	88.3 (882/999)§	52 (461/886)§	66.2 (524/791)§
# of all positive bc during phase, #	49	173	96	146
relative # of positive bc during phase, # (%)	19.6 (49/250)	19.6 (173/882)*	20.8 (96/461)	27.9 (146/524)*
# of positive bc without CNS during phase, #	24	131	52	80
relative # of positive bc without CNS during phase, # (%)	9.6 (24/250)	14.9 (131/882)*	11.3 (52/461)	15.3 (80/524)*

Outcome of patients enrolled (*n *= 999) with severe sepsis or septic shock is shown corresponding to the distinct phases as defined in Figure 1, starting with phase 0 including a period of 10 days prior to diagnosis of sepsis up to the end of phase III at Day 150. Also, the absolute numbers of drawn blood culture samples, the rate of blood culture samples per number of patients, the rate of all positive blood cultures (including CNS) and the rate of positive blood cultures without CNS are presented. CNS, Coagulase negative staphylococci; §, indicates statistically significant difference between all phases (*P *≤ 0.05; χ^2 ^test); *, indicates statistically significant difference compared to phase 0 and II (*P *≤ 0.05; χ^2 ^test); #, absolute numbers; %, relative number; bc, blood cultures; wrt, with respect to.

### Positive blood cultures without CNS

In total, 2,117 blood cultures were drawn. Prior to diagnosis in phase 0, 250 blood cultures were obtained, equaling a sampling rate of 25%, of which 9.6% were positive. During phase I, 882 blood cultures were drawn, representing a sampling rate of 88.3% with a positive rate of 14.9%. For the 886 patients alive in phase II, 461 samples were taken indicating a sampling rate of 52% and a positive rate of 11.3%. In the last phase III from Day 16 until Day 150, 524 samples were obtained representing a sampling rate of 66.2% with a positive rate of 15.3% (Table 2). There was a significant reduction in the numbers of drawn specimens per patient in phase II (phase II vs. phase I *P *≤ 0.01; χ^2 ^test) and a significant re-increase in phase III (phase III vs. phase II *P *≤ 0.01; χ^2 ^test). A significant increase in the rate of positive blood cultures was found in phase I compared to phase 0 (*P *≤ 0.05; χ^2 ^test), the rate of positive findings dropped in phase II (*P *≤ 0.05; χ^2 ^test) and rose again in phase III (*P *≤ 0.05; χ^2 ^test) comparable to levels of phase I (*P *> 0.05; χ^2 ^test).

### Epidemiology of microorganisms

A detailed overview of the epidemiology of isolated microorganisms depicted according to the different phases is presented in the online data supplement and a short overview in Table 3 (see also, Additional file 1). The rate of bacteria classified as typically opportunistic bacteria (TOB) increased over time from 7.7% in phase 0 up to 17.8% in phase III. In phase III, TOB were significantly more often identified in comparison to phase 1 (phase I vs. phase III *P *≤ 0.05; χ^2 ^test). Furthermore, the rate of isolated *Candida *spp. increased from 7.7% in phase 0 up to 35.7% in phase II and 30% in phase III. Both later phases were characterized by a significantly higher rate of *Candida *spp. compared to both earlier phases (phase II or III vs. phase 0 or I; *P *≤ 0.05; χ^2 ^test). The rate of CNS ranged from 50.9% in phase 0 to 26.7% in phase I (phase I vs. phases 0, II, III; *P *≤ 0.01; χ^2 ^test).

**Table 3:** Epidemiology of isolated microorganisms

microorganisms	phase 0	phase I	phase II	phase III
number of positive bc without CNS, #	26	143	56	90
typically opportunistic bacteria (TOB), % (#)	7.7 (2)	9.1 (13)	14.3 (8)	17.8 (16)*
Candida *spp*. overall, % (#)	7.7 (2)	12.6 (18)	35.7 (19)§	30 (27)§
pathogenic bacteria, % (#)	88.5 (23)	78.3 (112)	51.8 (29)	52.2 (47)
CNS *ssp*. overall, #	28	52	53	78

Isolated microorganisms from blood cultures of patients with severe sepsis or septic shock are demonstrated. The absolute as well as relative numbers are given in dependency of defined phases. Others include all other isolated pathogen and presented separately in the online supplement (Additional file 1: Table S1). CNS are also presented but excluded from relative analyses. CNS, Coagulase negative staphylococci; bc, blood cultures; *, indicates statistically significant difference compared to phase I (*P *≤ 0.05; χ^2 ^test); §, indicates statistically significant difference compared to phase 0 and phase I (*P *≤ 0.05; χ^2 ^test).

### Severity and use of antimicrobials

In the severity of disease measured by SOFA score, as well as in the course of PCT as a prototypic biomarker of infection, significant differences were found between the various phases. In phase I, both parameters peaked with the highest levels (phase I vs. phases 0, II, III; *P *≤ 0.01; ANOVA, Table 4) and fell over time to reach the lowest levels at phase III (phase III vs. phases 0, I, II; *P *≤ 0.01; ANOVA). The same was true for the requirement of mechanical ventilation with the highest rate of 90.9% in phase I (phase I vs. phases 0, II, III; *P *≤ 0.01; χ^2 ^test). In the use of renal replacement therapy with exception of phase 0, in which only 13% and, therefore, significantly fewer patients needed dialyses, no significant differences between the phases were found (phase 0 vs. phases I, II, III; *P *≤ 0.01; ANOVA).

**Table 4:** Patients characteristics and the use of antimicrobials

phases in relation to diagnosis	phase 0		phase I		phase II		phase III	
**Overall**								
# of patients on ICU during phase, #	661		999		690		327	
SOFA Score, mean ± SD	8.5 ± 3.6		9.6 ± 3.8*		8.4 ± 4.0		7.1 ± 3.9	
PCT, mean ± SD	5.0 ± 10.8		10.3 ± 23.5*		3.2 ± 8.5		2.0 ± 4.9	
Requirement of mechanical ventilation, % (#)	78.8 (521)		90.9 (908)*		82.8 (571)		83.8 (274)	
Requirement of renal replacement therapy, % (#)	13.2 (87)§		27.7 (277)		28.7 (198)		30.9 (101)	
Treatment with antimycotica, % (#)	5.1 (34)^2^		10.6 (106)^2^		17.5 (121)^2^		28.7 (94)^2^	
*Amphotericin*	2.6 (17)		3 (30)		4.8 (33)		8 (26)	
*Fluconazole/Itroconazole/Ketoconazole*	2 (13)		5.3 (53)		9.6 (66)		20.8 (68)	
*Voriconazole*	1.1 (7)		1.8 (18)		4.1 (28)		5.2 (17)	
*Echinocadine (Anidulafungin/Caspofungin)*	0.3 (2)		1.3 (13)		1.9 (13)		3.4 (11)	
Treatment with antibiotica, % (#)	75.2 (497)^2^		97.5 (974)^2^		91.6 (632)^2^		81.3 (266)^2^	
*Glycopeptides (Teichoplanin/Vancomycine)*	10.3 (68)		19.2 (192)		31.3 (216)		34.3 (112)	
*aminoglycosides*	5.3 (35)		7.9 (79)		7.5 (52)		10.7 (35)	
*Glycylcyclines*	1.2 (8)		1.8 (18)		4.6 (32)		11.3 (37)	
*quinolones*	11.8 (78)		18.1 (181)		24.8 (171)		38.2 (125)	
*carbapenems*	15.8 (104)		35.2 (352)		48.1 (332)		40.1 (131)	
*cephalosporins (3rd/4th generation)*	11.8 (78)		14.4 (144)		14.3 (99)		20.2 (66)	
*tetracyclines*	0 (0)		0.2 (2)		0.1 (1)		0.3 (1)	
*ansamycins*	2 (13)		2.7 (27)		2.8 (19)		3.4 (11)	
*oxazolidinones*	1.4 (9)		2.4 (24)		3.5 (24)		8 (26)	
*penicillin*	39.2 (259)		57.1 (570)		39.9 (275)		27.5 (90)	
*others+*	31.3 (207)		29.5 (295)		28.4 (196)		27.2 (89)	
**In dependency of bloodculture results**	bc -	bc +	bc -	bc +	bc -	bc +	bc -	bc +
SOFA Score, mean ± SD	9.19 ± 3.93	9.29 ± 4.02	10.02 ± 3.58	10.52 ± 3.67	9.58 ± 3.7	10.18 ± 4.53	8.7 ± 3.51	8.42 ± 4.07
PCT, mean ± SD	5.24 ± 7.38	8.1 ± 11.34	12.12 ± 25.25	20.86 ± 43.82³	3.09 ± 7.82	6.68 ± 12.32³	2.07 ± 4.12	4.89 ± 12.5³
Requirement of mechanical ventilation, % (#)	76.4 (120/157)	100 (7/7)	79.3 (521/657)	95.4 (103/108)³	66 (210/318)	92.5 (37/40)³	44.8 (126/281)	79.2 (38/48)³
Requirement of renal replacement therapy, % (#)	85.7 (36/42)	100 (2/2)	84.9 (141/166)	100 (30/30)³	81.8 (54/66)	90.9 (10/11)	48.2 (39/81)	85.7 (18/21)³

Selected characteristics and antimicrobial treatment of patients corresponding to the distinct phases are demonstrated. The absolute, relative numbers and mean ± SDs are given. CNS, coagulase negative staphylococci; +, others include cephalosporins of the first and second generation, lincosamides, folic acid antagonists, macrolide antibiotics and nitroimidazole; #, absolute numbers; %, relative number; bc, blood cultures; *, indicates statistically significant difference compared to phase 0, phase II and phase III (*P *≤ 0.05; ANOVA and χ^2 ^test); §, indicates statistically significant difference compared to phase I, phase II and phase III (*P *≤ 0.01; ANOVA); ^2^, indicates statistically significant differences between all phases (*P *≤ 0.05; ANOVA); ³, indicates statistically significant difference compared to bc^- ^(*P *≤ 0.05; ANOVA and χ^2 ^test).

The rates of antimycotic therapy increased significantly over time with the highest rate of 28.7% during phase III (phase III vs. phases 0, I, II; *P *≤ 0.01; χ^2 ^test). Azole antifungal drugs were prescribed most often. Antibiotic treatment was performed most often in phase I with a rate of 97.5% (phase I vs. phases 0, II, III; *P *≤ 0.01; χ^2 ^test). The primarily used antibiotics during this time were carbapenems and glycopeptides.

Additional analyses, referring to the various phases and results of blood cultures were performed, including the SOFA score, PCT levels and the requirement of mechanical ventilation and renal replacement therapy on the day the corresponding blood culture was sampled. No significant differences were found in the SOFA score. In contrast, higher PCT levels and rates of mechanical ventilations were found in patients with positive blood cultures during observation phase I, phase II and phase III (bc^- ^vs. bc^+ ^in phases I, II, III; *P *≤ 0.05; χ^2 ^test and ANOVA). For the use of renal replacement therapy this was true for phase I and III (bc^- ^vs. bc^+ ^in phases I, III; *P *≤ 0.05; χ^2 ^test).

## Discussion

Our findings demonstrate in a large retrospective clinical study, the presence of three distinct mortailty peaks and two nadirs in the course of the monitored mortality rates. We were, therefore, able to identify three clinical phases, phase I - approximately until Day 5 after the onset of sepsis, phase II - the following days until Day 15, and phase III - beginning at Day 16, strictly determined by such peaks and nadirs. A total of 63.3% of all deaths occurred during the defined late phases II and III, which implicates a broad clinical significance. Furthermore, our data demonstrate a significantly renewed rate of positive blood cultures occurring in phase III of sepsis, as well as the highest rates of TOB in this phase. High positive rates of blood cultures, and especially the rise of TOB during this phase, may indicate either an inability to overcome underlying infections or a possible increase in secondary infections. The rate of drawn blood culture samples is comparable overall to data from available literature and representative of practice patterns [14,15]. However, we observed an expected and significant reduction in the numbers of drawn specimens per patient in phase II and, surprisingly, a significant increase in phase III.

The rates of positive cultures ranged from 9.6% prior to diagnosis, significantly up to 14.9% during phase I, dropping during phase II to levels of 11.3% and rising again to 15.3% during the last observational period - phase III. These significant patterns of positive findings and the increase of TOB from 9.1% in phase I up to 17.8% in phase III might indicate an inability to clear persistent infections or the appearance of new, secondary infections, and potentially suggest the clinical significance of the late phase of sepsis. It might be speculated that one of the underlying reasons could be the proposed immune compromised status of patients with sepsis developing during the progression of disease. Additionally, the rate of *Candida *spp. significantly increased from 12.6% in phase I up to 35.7% in phase II, and to 30.0% in phase III. An underlying mechanism might be the frequent use of antibiotics in phase I.

In this retrospective review, we attempt to explore the clinically impacted and microbiological pattern of the late phase of sepsis as a cause of late morbidity and mortality by dividing sepsis, according to the observed mortality peaks, into three periods. We detected a significant increase of positive blood cultures drawn in patients with sepsis at a later time point with higher rates of TOB, which may indicate an anergic immune system. It needs to be understood that such implications have a number of important drawbacks due to the design of this study. First, the demonstrated higher frequency rate of positive cultures will be very sensitive to existing practice patterns. In our ICU the decision to draw cultures will be taken according to the guidelines recommending a sampling by signs and symptoms of infections as a new onset of fever, chills, hypothermia, leukocytosis, PCT (demonstrated by the high levels in bc^+/- ^patients at the sampling day) or a raise in CRP, neutropenia or left shift in differential blood count. In our opinion, sampling based on guidelines without a prospective investigative protocol reflects the situation on ICU more reliably. The sampling rate clearly shows a drop from 88.3% in phase I to 52% in phase II and a rise up to 66.2% in phase III. This might demonstrate the still ongoing inflammatory response in these patients, especially after phase I and II in which almost all of our patients had already received guideline-appropriate antibiotic treatment. In contrast to the early phase, in which well described guidelines for the sampling indication and procedure exist, the indication for blood culture sampling during the late time points are often also determined by the clinical phenotype and may not be as precisely captured when compared to the onset phase. This fact represents a confounder which is very difficult to control. Within this study, we used a definition of TOB. However, a precise definition of TOB does not exist, and it must be pointed out that our classification was based on data from the literature and the expert opinion of microbiologists and intensive care physicians, which may also represent a drawback with respect to derived implications. A further limitation of this study might be that there is important informative censoring going on; patients who die early contribute to the initial blood culture sampling quota but obviously not to the later ones. In contrast, patients who did well and left the ICU and were sampled later were still included in our analyses. Patients who did well and left the hospital were not observed further. However, all three scenarios provoke no bias for the rate of positive blood cultures. Also limiting might be the fact that the number and the duration of uses of a central venous catheter were not included in our analyses. Only the requirement of mechanical ventilation and renal replacement therapy as additional risk factors for infections were analyzed in greater detail. This study shows that patients with sepsis treated on our ICU at a later time point (>16 days) were still or even more likely to exhibit a positive blood culture in comparison to earlier time points and that such patients are more prone to develop TOBs. Of course, these results are not necessarily a legitimate or validated proxy for an anergic immune system, but due to the absence of a clinically established and relevant indicator for immune suppression in daily practices, the data might serve as further hints for this hypothesis, which has been much more clearly described in models of experimental sepsis.

## Conclusions

Most of the adjunctive clinical trials that were conducted with the aim to treat sepsis focused on early and anti-inflammatory mechanisms abrogating hyper-inflammation, with most of them failing to deliver the anticipated results [16-22]. Only a few trials were designed with a strategy aimed at the later phases or at improving immune suppression [23-25]. However, both strategies have so far not resulted in new therapeutics capable of significantly improving outcomes of septic patients. Our data demonstrate that the later phase of sepsis is associated with a significant re-increase of positive blood culture results, especially opportunistic bacteria and fungi, and that a majority of all deaths occurred after Day 5. These data have a large potential impact on treatment, monitoring and outcome of septic patients. However, the underlying mechanisms are so far not well understood. It, therefore, appears to be warranted to pay more attention to the late phase of sepsis and to develop strategies detecting the patients' host immune response level.

## Key messages

• The time course of disease in a large population of patients suffering from severe sepsis or septic shock was characterized by three different mortality peaks, which suggests that these patients undergo phases which may also be dominated by different underlying pathophysiological mechanisms.

• The late phase of sepsis is characterized by a significant resurgence of positive blood culture findings, which underlines the importance of the innate immune responses in this phase.

• Positive microbiological findings of opportunistic bacteria and *Candida *spp. increase over the length of ICU stay in patients with severe sepsis and septic shock.

• In sepsis, diagnostic tools monitoring the immune status as well as therapies aimed at restoring the immune response are urgently needed.

## Abbreviations

#: numbers; *: indicates statistically significant differences P ≤0.05; %: relative number; χ^2^: Chi-square test; abs: absolute numbers; bc: blood cultures; bc^+/-^: positive or negative blood cultures; CNS: coagulase negative staphylococci; PCT: procalcitonin; SOFA score: Sequential Organ Failure Assessment score; TOB: typically opportunistic bacteria; wrt: with respect to.

## Competing interests

The authors declare that they have no competing interests.

## Authors' contributions

GPO designed the study and performed data analysis and interpretation, and wrote the first draft of the manuscript. MS, RAC and KM were involved in data analysis and interpretation, and the writing of the manuscript. JR was involved in microorganism identification and interpretation. KR was involved in study design and data collection. MB was involved in data management and interpretation. NCR was involved in study design, data analysis, and the writing of the manuscript. All authors read and approved the final draft of the manuscript.

## Supplementary Material

Additional file 1 **Additional Table S1. Supplement **Table 1**Epidemiology of isolated microorganisms**. Isolated microorganisms from blood cultures of patients with severe sepsis or septic shock are listed. The absolute as well as relative numbers are given dependent on the pre-defined phases. CNS are also presented but excluded from relative analyses. Click here for file
