# Supplementary material for: The late phase of sepsis is characterized by an increased microbiological burden and death rate
Source: Crit Care. 2011 Jul 28;15(4):R183. doi: 10.1186/cc10332 (PMC3387626; doi:10.1186/cc10332)
Supplement: Additional file 1 — Additional Table S1. Supplement Table 1Epidemiology of isolated microorganisms. Isolated microorganisms from blood cultures of patients with severe sepsis or septic shock are listed. The absolute as well as relative numbers are given dependent on the pre-defined phases. CNS are also presented but excluded from relative analyses. [file cc10332-S1.DOC]

Supplement Table 1 Epidemiology of isolated microorganisms

|  |  |  |  |  |
| --- | --- | --- | --- | --- |
| **microorganisms** | **phase 0** | **phase I** | **phase II** | **phase III** |
| **number of positive bc, #** | **26** | **143** | **56** | **90** |
| **typically opportunistic bacteria (POB), % (#)** | **7.7 (2)** | **9.1 (13)** | **14.3 (8)** | **17.8 (16)*** |
| Acinetobacter baumannii |  | 0.7 (1) |  |  |
| Corynebacterium jeikeum |  |  | 1.8 (1) |  |
| Corynebacterium macginleyi |  |  | 1.8 (1) |  |
| Enterobacter aerogenes |  | 1.4 (2) | 1.8 (1) | 1.1 (1) |
| Eubacterium lentum |  |  | 1.8 (1) |  |
| Klebsiella pneumoniae ssp. pneumoniae | 3.8 (1) | 2.1 (3) | 1.8 (1) | 5.6 (5) |
| Leuconostoc spp. |  |  | 1.8 (1) |  |
| Pantoea agglomerans |  | 0.7 (1) |  |  |
| Peptostreptococcus |  |  |  | 1.1 (1) |
| Proteus mirabilis |  | 0.7 (1) |  | 2.2 (2) |
| Proteus vulgaris |  | 0.7 (1) |  |  |
| Pseudomonas aeruginosa | 3.8 (1) | 2.8 (4) | 3.6 (2) | 7.8 (7) |
|  |  |  |  |  |
| **Candida *spp.* overall, % (#)** | **7.7 (2)** | **12.6 (18)** | **35.7 (19)§** | **30 (27)§** |
| Candida albicans | 3.8 (1) | 5.6 (8) | 17.9 (10) | 25.6 (23) |
| Candida dubliniensis |  | 0.7 (1) |  |  |
| Candida glabrata |  | 2.1 (3) | 5.4 (3) | 1.1 (1) |
| Candida kefyr |  |  | 1.8 (1) |  |
| Candida globosa |  | 0.7 (1) |  |  |
| Candida krusei |  | 0.7 (1) |  |  |
| Candida parapsilosis |  | 1.4 (2) | 1.8 (1) | 1.1 (1) |
| Candida tropicalis | 3.8 (1) | 1.4 (2) | 1.8 (1) | 2.2 (2) |
| other Candidia ssp. |  |  | 1.8 (1) |  |
|  |  |  |  |  |
| **pathogenic bacteria, % (#)** | **88.5 (23)** | **78.3 (112)** | **51,8 (29)** | **52.2 (47)** |
| Bacteroides fragilis | 3.8 (1) | 0.7 (1) |  |  |
| Bacteroides thetaiotaomicron | 3.8 (1) |  |  |  |
| Brevibacterium spp. | 3.8 (1) |  |  |  |
| Clostridium clostridiiforme | 3.8 (1) |  |  |  |
| Corynebacterium striatum/amycolaticum |  |  | 1.8 (1) |  |
| Enterobacter cloacae | 3.8 (1) | 6.3 (9) | 1.8 (1) | 1.1 (1) |
| Enterobacter sakazakii |  | 0.7 (1) |  |  |
| Enterococcus avium | 3.8 (1) |  |  |  |
| Enterococcus casseliflavus |  | 0.7 (1) |  |  |
| Enterococcus faecalis | 7.7 (2) | 9.1 (13) | 7.1 (4) | 13.3 (12) |
| Enterococcus faecium | 15.4 (4) | 6.3 (9) | 23.2 (13) | 8.9 (8) |
| Enterococcus gallinarum |  |  | 1.8 (1) |  |
| Escherichia coli | 19.2 (5) | 18.2 (26) | 1.8 (1) | 8.9 (8) |
| Fusobacterium spp. |  | 0.7 (1) | 1.8 (1) |  |
| Beta-hemolytic Streptococcus group B | 7.7 (2) |  |  |  |
| Beta-hemolytic Streptococcus group G |  | 2.1 (3) |  |  |
| Klebsiella oxytoca |  | 3.5 (5) |  |  |
| Micrococcus luteus |  |  |  | 1.1 (1) |
| Prevotella spp. |  |  |  | 1.1 (1) |
| Propionibacterium |  | 2.1 (3) | 3.6 (2) | 5.6 (5) |
| Saccharomyces cerevisiae |  |  | 1.8 (1) |  |
| Serratia marcescens |  | 2.8 (4) | 1.8 (1) | 1.1 (1) |
| Staphylococcus aureus | 11.5 (3) | 21.7 (31) | 5.4 (3) | 10 (9) |
| Streptococcus anginosus |  | 0.7 (1) |  |  |
| Streptococcus bovis | 3.8 (1) |  |  |  |
| Streptococcus group C |  | 0.7 (1) |  |  |
| Streptococcus mitis/Streptococcus oralis |  | 0.7 (1) |  | 1.1 (1) |
| Streptococcus pneumoniae |  | 0.7 (1) |  |  |
| Streptococcus salivarius |  | 0.7 (1) |  |  |
|  |  |  |  |  |
| **CNS *spp*. overall, #** | **28** | **52** | **53** | **78** |
| Staphylococcus capitis | 1 |  |  | 1 |
| Staphylococcus epidermidis | 23 | 39 | 43 | 60 |
| Staphylococcus haemolyticus | 2 | 7 | 3 | 4 |
| Staphylococcus hominis | 2 |  | 3 | 7 |
| Staphylococcus kloosii |  |  |  | 1 |
| Staphylococcus lugdunensis |  | 1 |  |  |
| Staphylococcus saccharolyticus |  | 1 |  |  |
| Staphylococcus saprophyticus |  | 4 | 2 | 1 |
| Staphylococcus simulans |  |  | 1 | 4 |
| other Staphylococci ssp. |  |  | 1 |  |
|  |  |  |  |  |

Isolated microorganisms from blood cultures of patients with severe sepsis or septic shock are listed. The absolute as well as relative numbers are given dependent on the pre-defined phases. CNS are also presented but excluded from relative analyses. CNS, Coagulase negative staphylococci; bc, blood cultures; *, indicates statistically significant difference compared to phase I (p ≤ 0.05; χ^2^ test); §, indicates statistically significant difference compared to phase 0 and phase I (p ≤ 0.05; χ^2^ test).
